# Identification of BHLHE40 expression in peripheral blood mononuclear cells as a novel biomarker for diagnosis and prognosis of hepatocellular carcinoma

**DOI:** 10.1038/s41598-021-90515-w

**Published:** 2021-05-27

**Authors:** Pattapon Kunadirek, Chaiyaboot Ariyachet, Supachaya Sriphoosanaphan, Nutcha Pinjaroen, Pongserath Sirichindakul, Intawat Nookaew, Natthaya Chuaypen, Pisit Tangkijvanich

**Affiliations:** 1 Department of Biochemistry, Faculty of Medicine, Center of Excellence in Hepatitis and Liver Cancer, Chulalongkorn University, Bangkok, 10330 Thailand; 2 Division of Gastroenterology, Department of Medicine, Faculty of Medicine, Chulalongkorn University, Bangkok, 10330 Thailand; 3 Department of Radiology, Faculty of Medicine, Chulalongkorn University, Bangkok, 10330 Thailand; 4 Department of Surgery, Faculty of Medicine, Chulalongkorn University, Bangkok, 10330 Thailand; 5 Department of Biomedical Informatics, College of Medicine, University of Arkansas for Medical Sciences, Little Rock, 72205 USA

**Keywords:** Cancer, Molecular biology, Biomarkers

## Abstract

Novel and sensitive biomarkers is highly required for early detection and predicting prognosis of hepatocellular carcinoma (HCC). Here, we investigated transcription profiles from peripheral blood mononuclear cells (PBMCs) of 8 patients with HCC and PBMCs from co-culture model with HCC using RNA-Sequencing. These transcription profiles were cross compared with published microarray datasets of PBMCs in HCC to identify differentially expressed genes (DEGs). A total of commonly identified of 24 DEGs among these data were proposed as cancer-induced genes in PBMCs, including 18 upregulated and 6 downregulated DEGs. The KEGG pathway showed that these enriched genes were mainly associated with immune responses. Five up-regulated candidate genes including BHLHE40, AREG, SOCS1, CCL5, and DDIT4 were selected and further validated in PBMCs of 100 patients with HBV-related HCC, 100 patients with chronic HBV infection and 100 healthy controls. Based on ROC analysis, BHLHE40 and DDIT4 displayed better diagnostic performance than alpha-fetoprotein (AFP) in discriminating HCC from controls. Additionally, BHLHE40 and DDIT4 had high sensitivity for detecting AFP-negative and early-stage HCC. BHLHE40 was also emerged as an independent prognostic factor of overall survival of HCC. Together, our study indicated that BHLHE40 in PBMCs could be a promising diagnostic and prognostic biomarker for HBV-related HCC.

## Introduction

Hepatocellular carcinoma (HCC), one of the malignant tumors with high heterogeneity, is a leading cause of cancer-related deaths worldwide, especially where chronic hepatitis B virus (HBV) infection is common^1^. Accurate assessment of HCC risk at the early state is very important to optimize the opportunity in receiving curative health care interventions, such as surgical and ablative therapies. In addition, the prognosis of patients with HCC remains unsatisfactory due to the aggressiveness and high recurrence rates of the cancer^1^. At present, alpha‐fetoprotein (AFP), a fetal-specific glycoprotein is the most widely used biomarker for HCC screening. Despite its routine use in clinical practice, AFP provides a low sensitivity of 58–68% in detecting an early-stage HCC and its level might be elevated in non-malignant chronic liver disease^2^. Therefore, obtaining reliable serum biomarkers for early diagnosis and prognostic prediction are highly required to improve the outcome and overall survival of patients with HCC.

Biomarkers produced by cancer cells including altered gene expression and methylation could be identified in the adjacent body fluid or blood circulation, leading to a new approach for a minimally invasive early cancer detection^3,4^. Indeed, growing evidence has revealed an important role of peripheral blood mononuclear cells (PBMCs) as novel circulating sources that are closely correlated with the pathogenesis of various malignancies^5^. In this context, recent studies demonstrated that changes of gene expression and methylation profiles in PBMCs were observed in patients with non-small cell lung cancer, renal cell carcinoma and breast cancer^6–8^. Regarding HCC, it was shown that the expression profiles of PBMCs differed significantly between patients with or without cancer and could be used as a surrogate approach for the assessment of tumor infiltrating lymphocytes^9^. In a previous study, a co-culture model was performed to investigate the alteration of PBMCs in HCC and identified the alteration of checkpoint inhibitor marker on PBMCs as a prognostic marker for HCC^10^. In addition, gene profiling in PBMCs detected by RNA-sequencing (RNA-Seq) could provide a potential tool for the diagnosis of advanced HCC with metastasis^11^. Together, these data have indicated that a co-culture model might be used to mimic the interaction of cancer and PBMCs, and this may access and identify altered genes in PBMCs from communicating with HCC as a useful marker in the detection and prognostication of HCC.

In this study, we examined whether secretion from cancer cells could induce gene expression in circulating WBCs of patients with HCC. To this end, we investigated transcriptional profiles of PBMCs derived from co-culture model to identify differential genes and cross comparison with previous studies^12^, resulted novel diagnostic biomarkers. The performance of these biomarkers was further validated in PBMCs of patients with HBV-related HCC in comparison with non-cancer controls by qRT-PCR. Finally, the prognostic role of these candidate genes in terms of overall survival of patients with HCC was also investigated.

## Results

### Integrated gene expression analysis of PBMCs

**Figure 1:**
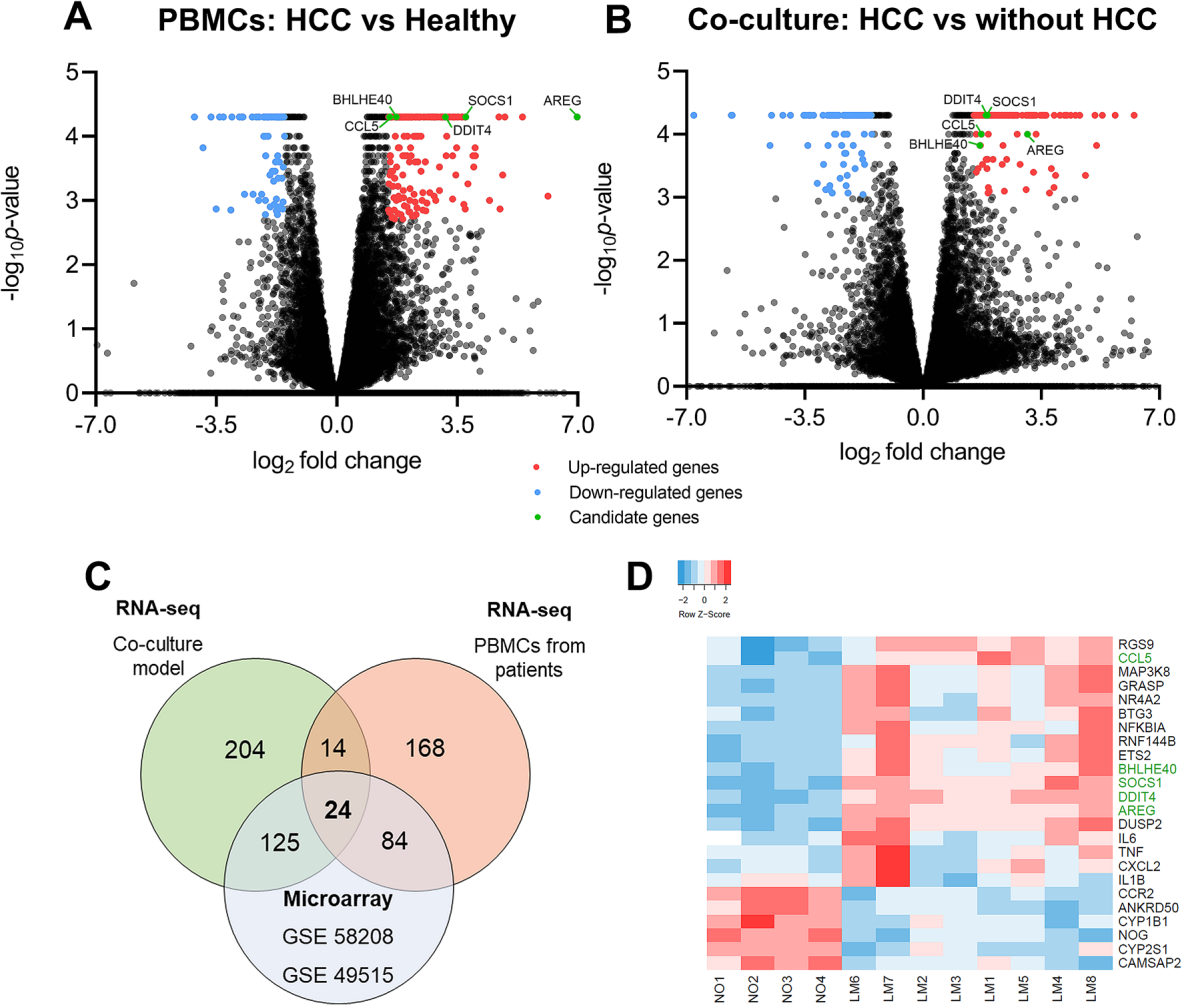
Transcriptome profiling of cancer-induce genes and integration transcription profiles of PBMCs. (**A**) volcano plot presents differentially expressed genes in PBMCs compared with healthy controls. (**B**) Volcano plot presents differentially expressed genes in PBMCs of co-culture compared with control. Volcano plot shows − log_10_*P*-values on the y-axis and fold change expressed as log_2_ on the x-axis. Red scatter dots represent up-regulated genes with *P* ≤ 0.05 and log_2_fold change ≥ 1.5. Blue scatter dots represent down with *P* ≤ 0.05 and log2fold change ≤ -1.5. Green scatter dots represent a candidate of cancer-induced genes for validation in PBMCs. (**C**) Venn diagram represents intersect genes between 3 transcription profiles of PBMCs including PBMCs from patients with HCC, PBMCs from co-culture model and published microarray data. (**D**) Heatmap of cancer-induced genes expression in PBMCs of patients with HCC. Candidate of cancer-induced genes for validation are labels in green.

To investigate the transcription profiles, PBMCs from 8 patients with HCC and 4 healthy controls, as well as PBMCs from 3 healthy controls co-cultured with HCC cancer cells (Huh7) were collected to perform RNA-Seq. Baseline characteristics of patients and healthy controls were presented in Supplementary Table S1. Our results showed that a total of 290 genes were identified as differentially expressed genes (DEGs) in PBMCs of patients with HCC compared with healthy controls, which included 213 up-regulated (*P* < 0.05, log_2_FC > 1.5) and 77 down-regulated genes (*P* < 0.05, log_2_FC < 1.5; Fig. 1A). In co-culture of PBMCs with Huh7 cells, we found a total of 367 DEGs with 222 up-regulated genes and 145 down-regulated genes in PBMC co-culture with HCC compared with PBMCs without HCC (Fig. 1B).

To identify whether the DEGs of RNA-Seq data from PBMCs of HCC represent as cancer-induced genes, DEGs from patients with HCC and co-culture model were compared with published microarray data sets (GSE 58208 and 49519)^12^ using Connection Up-and Down-Regulation Expression Analysis of Microarrays eXtension (CU-DREAMX)^13^. The intersection among three data sets were shown in Venn diagram (Fig. 1C). Our results showed that a total of 24 DEGs with 18 up-regulated and 6 down-regulated genes were overlapped among these data (Fig. 1C), and intersected genes across data were listed in Supplementary Table S2. To assess whether the above-mentioned 24 intersected genes differed significantly between patients with HCC and healthy controls, a hierarchical clustering using heatmap analysis was further performed. In this respect, the results demonstrated that these two groups could be clearly discriminated by these genes (Fig. 1D). In addition, heatmap analysis of 24 intersected genes in PBMCs from co-culture model and published microarray data were shown in Supplementary Fig. S1. Together, these 24 DEGs in PBMCs were speculated as cancer-induced genes due to the alteration of genes when interacting with HCC in the co-culture model and presenting in PBMCs of patients with HCC.

### Functional gene annotation and pathway enrichment analysis

**Figure 2:**
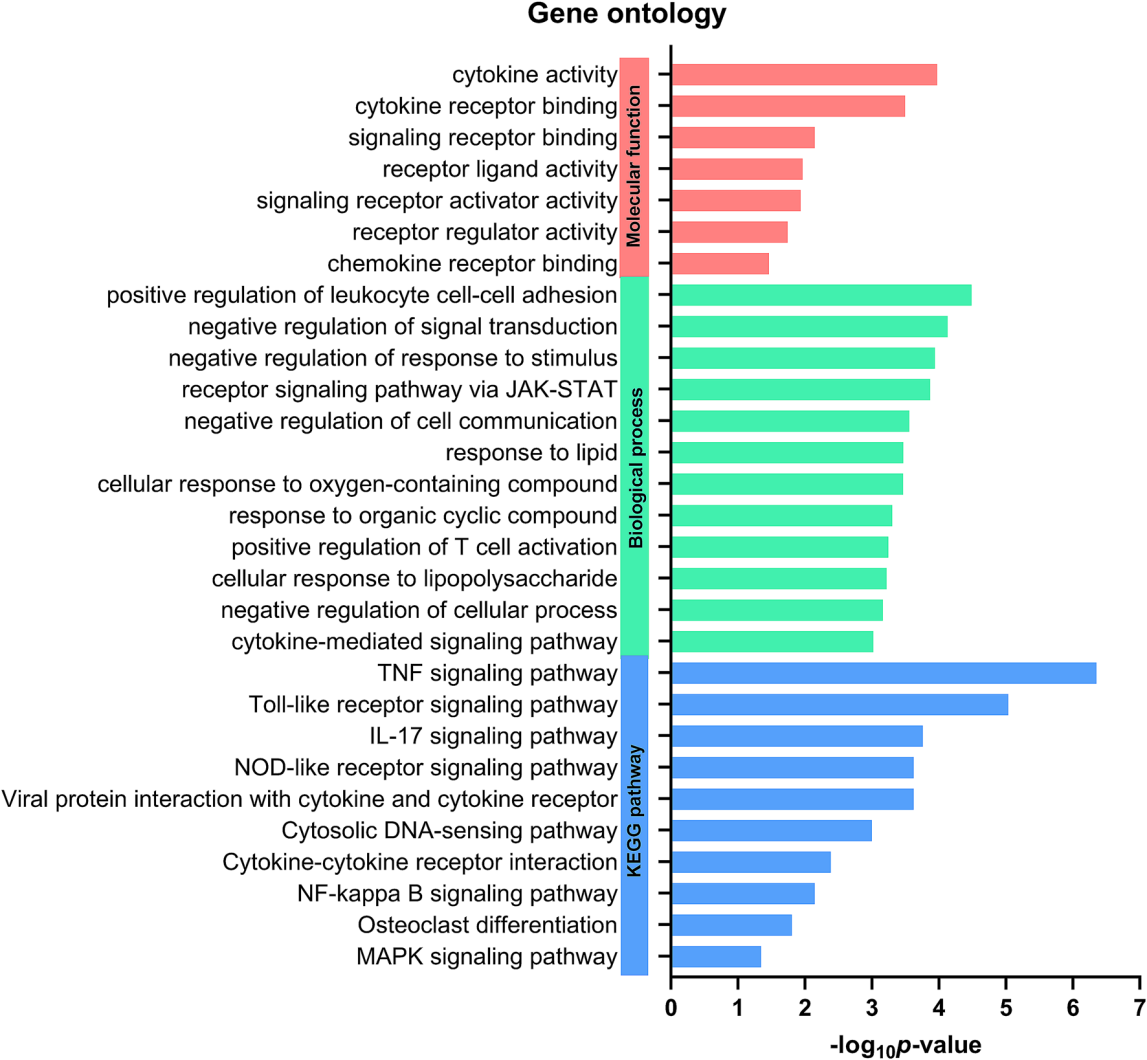
Gene Ontology (GO) analysis. Cancer-induced genes show molecular pathways and immune response patterns. Enriched molecular function, biological process and KEGG pathways with a *p* ≤ *0.05* are displayed.

Functional enrichment analyses have shown to play an important role in the identification of biological characteristics in transcriptome data. In this study, we performed Gene Ontology (GO) and gProfiler analysis to identify the functional and signaling pathway of DEGs. The majority of DEGs were significantly enriched in the molecular function such as cytokine activity and biological process such as positive regulation of leucocyte cell–cell adhesion, which involved in immune regulation (Fig. 2). Furthermore, enriched Kyoto Encyclopedia of Genes and Genomes (KEGG) pathways were similarly involved in immunological response, which included tumor necrosis factor (TNF), pro-inflammatory interleukin (IL)-17 and toll-like receptor signaling pathways.

### Selection and validation of candidate biomarkers in PBMCs of HCC patients

To investigate whether the identified DEGs in PBMCs could be used as biomarkers in clinical setting, 5 upregulated candidate genes that were consistently up-regulated across PBMCs samples in our RNA-seq analysis were selected. These genes including BHLHE40, AREG, SOCS1, CCL5 and DDIT4 (green labelled in Fig. 1A,B,D) were chosen for validation by qRT-PCR method. The validated cohort consisted of 100 patients with HBV-related HCC, 100 patients with chronic hepatitis B (CHB) and 100 healthy controls. Baseline characteristics in each group were shown in Table 1. Among these individuals, healthy controls and the non-HCC group were matched for age and gender with patients with HCC. Patients with HCC had significantly higher in serum albumin, aspartate aminotransferase (AST), alkaline phosphatase and AFP levels than patients without HCC. Additionally, patients with HCC had a higher proportion of cirrhosis and lower levels of platelet count compared with the non-HCC group. There was no significant difference between groups in terms of serum alanine aminotransferase (ALT) and total bilirubin levels.

**Table 1:** Baseline characteristic of the validation cohort.

Baseline characteristics	Healthy controls (n = 100)	Patients without HCC (n = 100)	Patients with HCC (n = 100)	*P*
Age (years)	56.3 ± 3.4	56.7 ± 8.4	57.7 ± 8.6	0.053
Gender (Male)	80 (80.0)	85 (85.0)	85 (85.0)	0.549
Total bilirubin (mg/dL)		0.9 ± 0.7	1.0 ± 0.7	0.729
Serum albumin (g/dL)		4.1 ± 0.6	3.6 ± 0.5	< 0.001*
Aspartate aminotransferase (IU/L)		36.6 ± 30.2	64.5 ± 53.5	< 0.001*
Alanine aminotransferase (IU/L)		42.9 ± 42.3	44.8 ± 25.8	0.709
Alkaline phosphatase (IU/L)		77.3 ± 50.6	125.3 ± 70.8	< 0.001*
Platelet count (10^9^/L)		223.2 ± 74.6	176.0 ± 93.9	0.002*
Alpha fetoprotein (ng/mL)		8.5 ± 13.9	6928.1 ± 28,444.2	0.016*
Presence of cirrhosis		11 (11.0)	80 (80.0)	< 0.001*
BCLC stage (0-A/B/C)		-	35(35.0)/50(50.0)/15(15.0)	-

**Figure 3:**
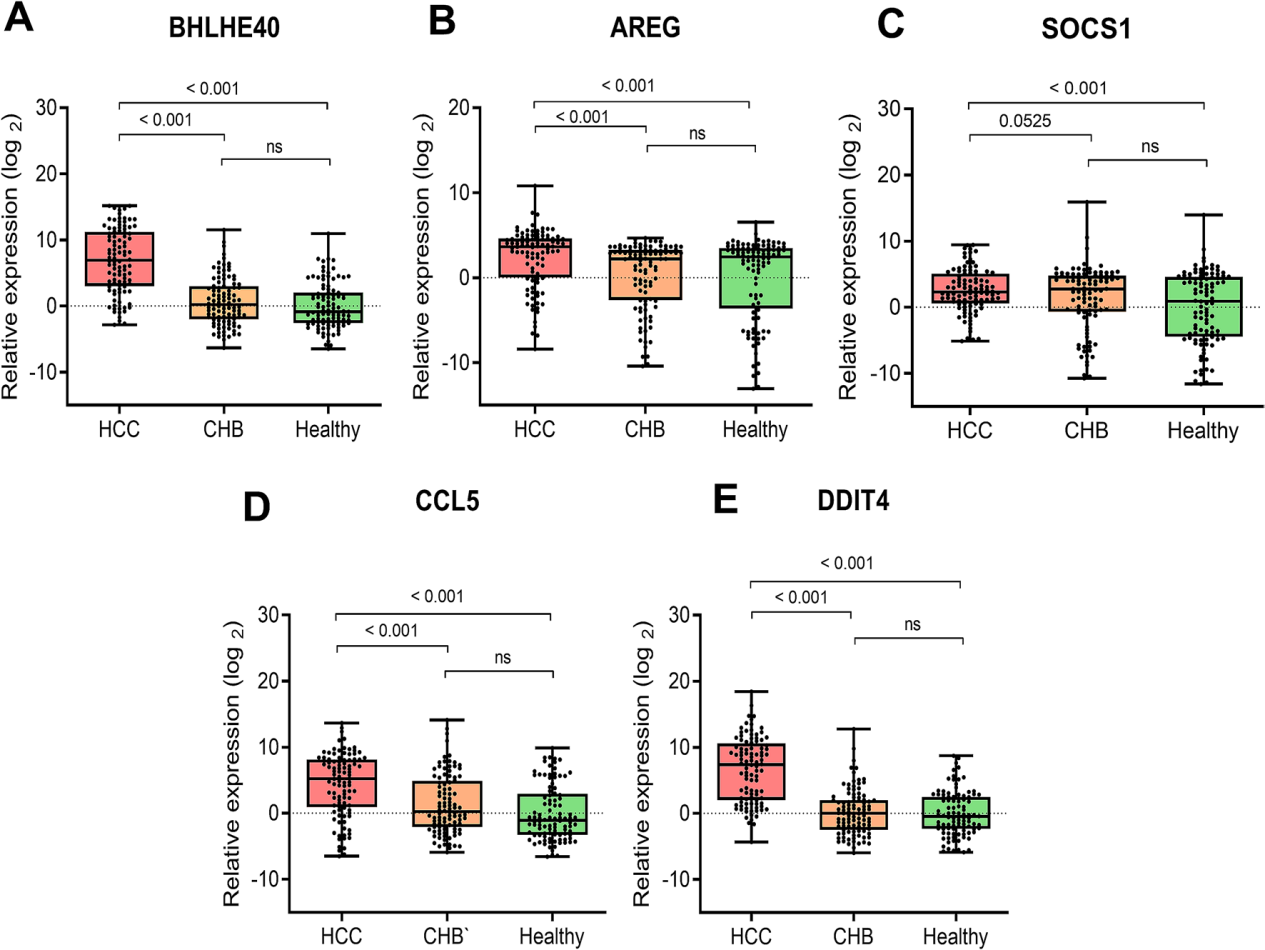
Relative expression of candidate cancer-induced genes in PBMCs of patients with HBV-HCC, CHB and healthy controls. (**A**) BHLHE40, (**B**) AREG, (**C**) SOCS1, (**D**) CCL5 and (**E**) DDIT4. Relative expression of genes represents as log_2_ on y-axis.

Our results demonstrated that BHLHE40, AREG, SOCS1, CCL5 and DDIT4 expression in patients with HCC were higher than those detected in the non-HCC group and healthy controls (Fig. 3). The average relative expression level of BHLHE40 in the HCC group (6.86 ± 4.89) were significantly higher than the non-HCC group (0.62 ± 3.65, *P* < 0.001) and healthy controls (0.00 ± 3.44, *P* < 0.001). Similarly, AREG levels in the HCC group were significantly higher than the non-HCC group (2.34 ± 2.53 vs 0.41 ± 3.92, *P* < 0.001) and healthy controls (0.00 ± 4.88, *P* < 0.001). For SOCS1 expression level, there was significantly higher in patients with HCC than healthy controls (2.55 ± 3.28 vs. 0.00 ± 5.39, *P* < 0.001), but did not reach statistical significance when compared with the non-HCC group (1.13 ± 4.94, *P* = 0.053). For CCL5, the levels of this gene in the HCC group (4.32 ± 4.81) were significantly different from the non-HCC (1.25 ± 4.46, *P* < 0.001) and healthy controls (0.00 ± 4.19, *P* < 0.001). A similar trend was found for DDIT4 expression in the HCC group (6.62 ± 4.48) when compared with the non-HCC group (0.16 ± 3.42, *P* < 0.001) and healthy controls (0.00 ± 3.37, *P* < 0.001). There was no statistically significant difference of these 5 genes between patients without HCC and healthy controls.

In subgroup analysis, patients with CHB were divided into the cirrhotic (n = 11) and non-cirrhotic (n = 89) groups. In Supplementary Fig. S2, there was no significant difference in the expression of all studied genes between the cirrhotic and non-cirrhotic groups. Of note, the mean relative expression levels of BHLHE40 and DDIT4 in the HCC group were significantly higher than the cirrhotic group (*P* < 0.001). These data might indicate that the expression levels of BHLHE40 and DDIT4 in PBMCs could effectively distinguish HCC from cirrhosis.

### Selected candidate genes as diagnostic markers of HCC

**Figure 4:**
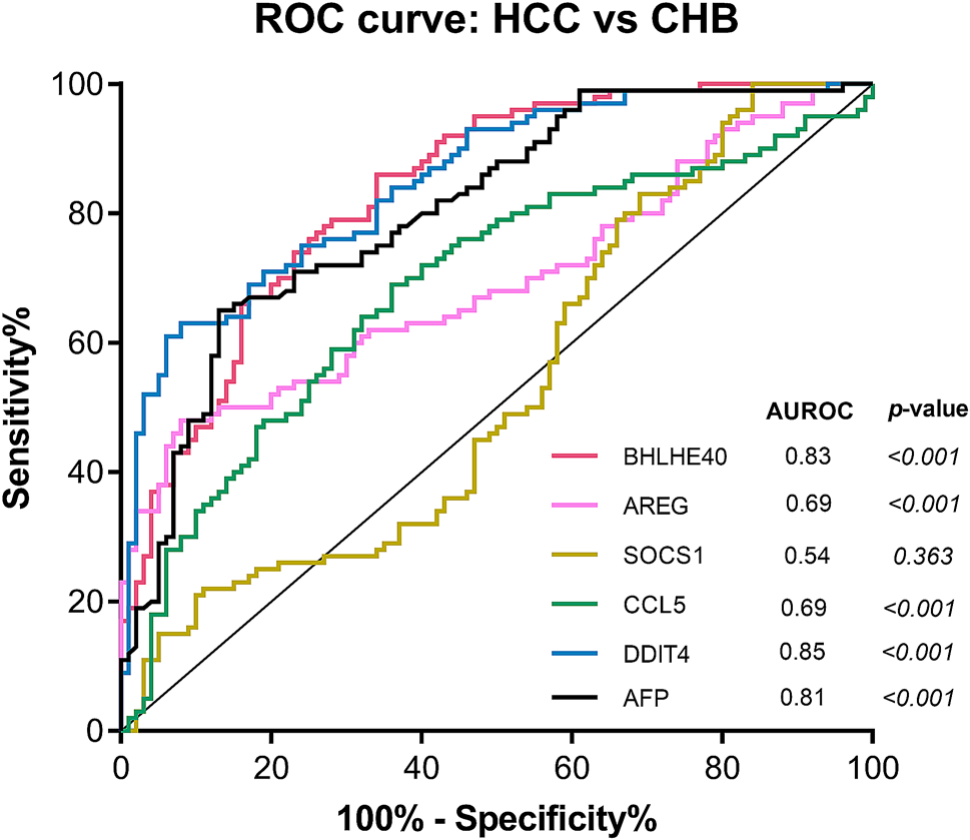
Receiver operating characteristic (ROC) curves of the cancer-induced genes of PBMCs in differentiating patients with HCC and non-HCC.

To investigate a diagnostic performance of candidate genes in discriminating HCC from non-HCC (including patients with CHB and healthy controls), the ROC curves were calculated. The area under the ROC curve (AUROC) was 0.83 [95% confidence interval (CI); 0.78–0.89, *P* < 0.001] for BHLHE40, 0.69 (95% CI 0.62–0.77, *P* < 0.001) for AREG, 0.54 (95% CI 0.46–0.62, *P* = 0.363) for SOCS1, 0.69 (95% CI 0.61–0.76, *P* < 0.001) for CCL5, 0.85 (95% CI 0.80–0.90, *P* < 0.001) for DDIT4 and 0.81 (95% CI 0.75–0.87, *P* < 0.001) for AFP (Fig. 4). The ROC curves of HCC vs healthy controls and CHB vs healthy controls were also analyzed (Supplementary Fig. S3). The data showed that there was a similar trend between HCC vs. non-HCC and HCC vs CHB. Additionally, the expression of these genes was not useful in distinguishing patients with CHB from healthy controls.

According to the ROC analysis, BHLHE40 and DDIT4 were considered the best biomarkers among the studied candidate genes, their diagnostic role was further assessed. In this respect, the optimal cut-off value of BHLHE40 in differentiating HCC from non-HCC was 1.80 with a sensitivity of 84.0% and specificity of 64.0%. Similarly, the cut-off value of DDIT4 was 2.10 with a sensitivity of 75.0% and specificity of 76.0%. The combination of BHLHE40 and DDIT4 without AFP slightly increased AUROC to 0.86 and improved accuracy to78.24% which was the highest accuracy in this study (Supplementary Table S3). Our results showed that BHLHE40 had the best sensitivity of 84% and AFP had highest specificity of 88%. Furthermore, CU-DREAMX analysis demonstrated that BHLHE40 and DDIT4 of PBMCs were not increased in patients with head and neck cancers and pancreatic cancer (Supplementary Fig. S4). These results suggested that BHLHE40 and DDIT4 could be used as potential biomarkers for HCC.

Based on their optimal cut-off values, the correlation of BHLHE40, AREG, SOCS1, CCL5 and DDIT4 expression with clinical parameters are shown in Supplementary Table S4.

### The diagnostic role of BHLHE40 and DDIT4 in AFP-negative HCC and small HCC

**Figure 5:**
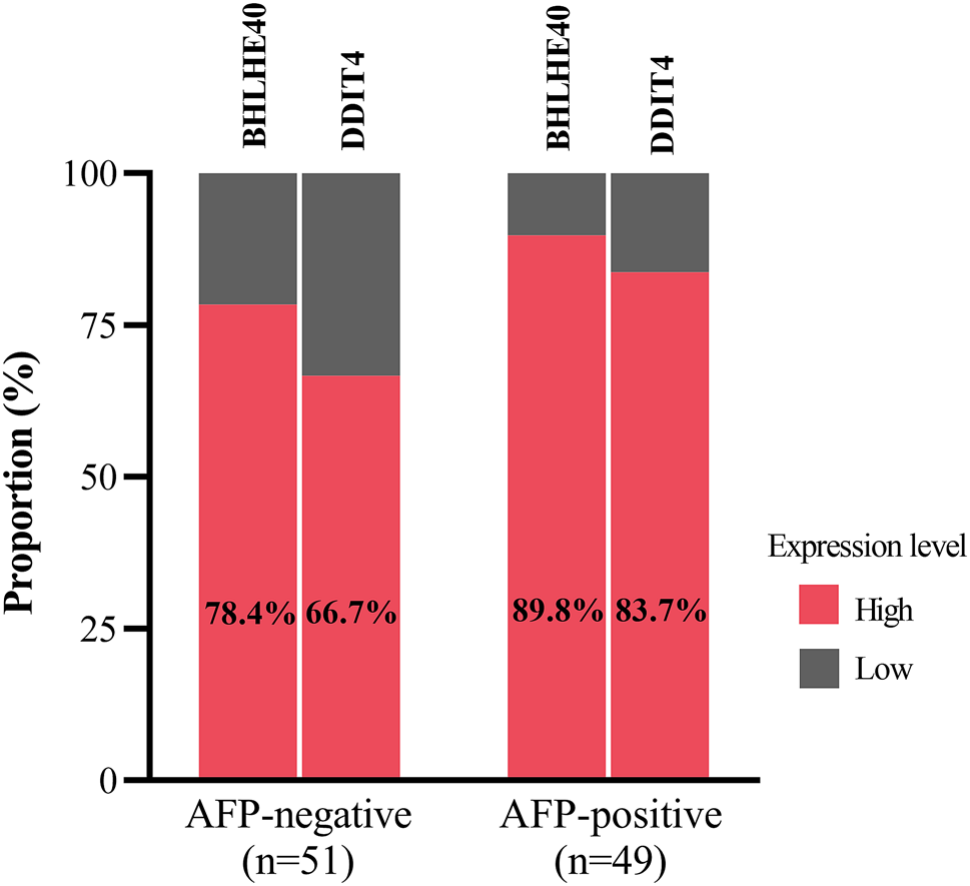
Proportion of BHLHE40 and DDIT4 expression in PBMCs of AFP- and AFP + patients with HBV-HCC.

Among patients with HCC, there was a strong correlation between BHLHE40 and DDIT4 (r = 0.826; *P* < 0.001). However, either BHLHE40 and DDIT4 was not correlated with AFP values (r = 0.197; *P* = 0.050 and r = 0.154; *P* = 0.126, respectively). Using the normal upper limit of AFP (20 ng/mL) as a reference, there were 51 (51%) and 49 (49%) patients with AFP-negative HCC and AFP-positive HCC, respectively (Fig. 5). Among the AFP-negative group, 78.4% (40/51) of patients had elevated BHLHE40 level (≥ 1.8) and 66.7% (34/51) of patients had high DDIT4 level (≥ 2.1). For the AFP-positive group, high levels of BHLHE40 and DDIT4 were detected in 89.8% (44/49) and 83.7% (41/49), respectively. Furthermore, 82.6% (42/51) of patients with AFP-negative HCC had elevated BHLHE40 and/or DDIT4 (Supplementary Fig. S5). Among early HCC (stages 0 and A), we found that 27.8% (10/36) patients had elevated AFP concentration, while 86.1% (31/36) and 69.4% (25/35) patients had elevated levels of BHLHE40 and DDIT4, respectively. Together, these results suggested that BHLHE40 and DDIT4 could be promising biomarkers for detecting AFP-negative HCC and early HCC, as well as they could be complementary to AFP in diagnosis of HCC.

### Prognostic performance of BHLHE40 and DDIT4 in patients with HCC

**Figure 6:**
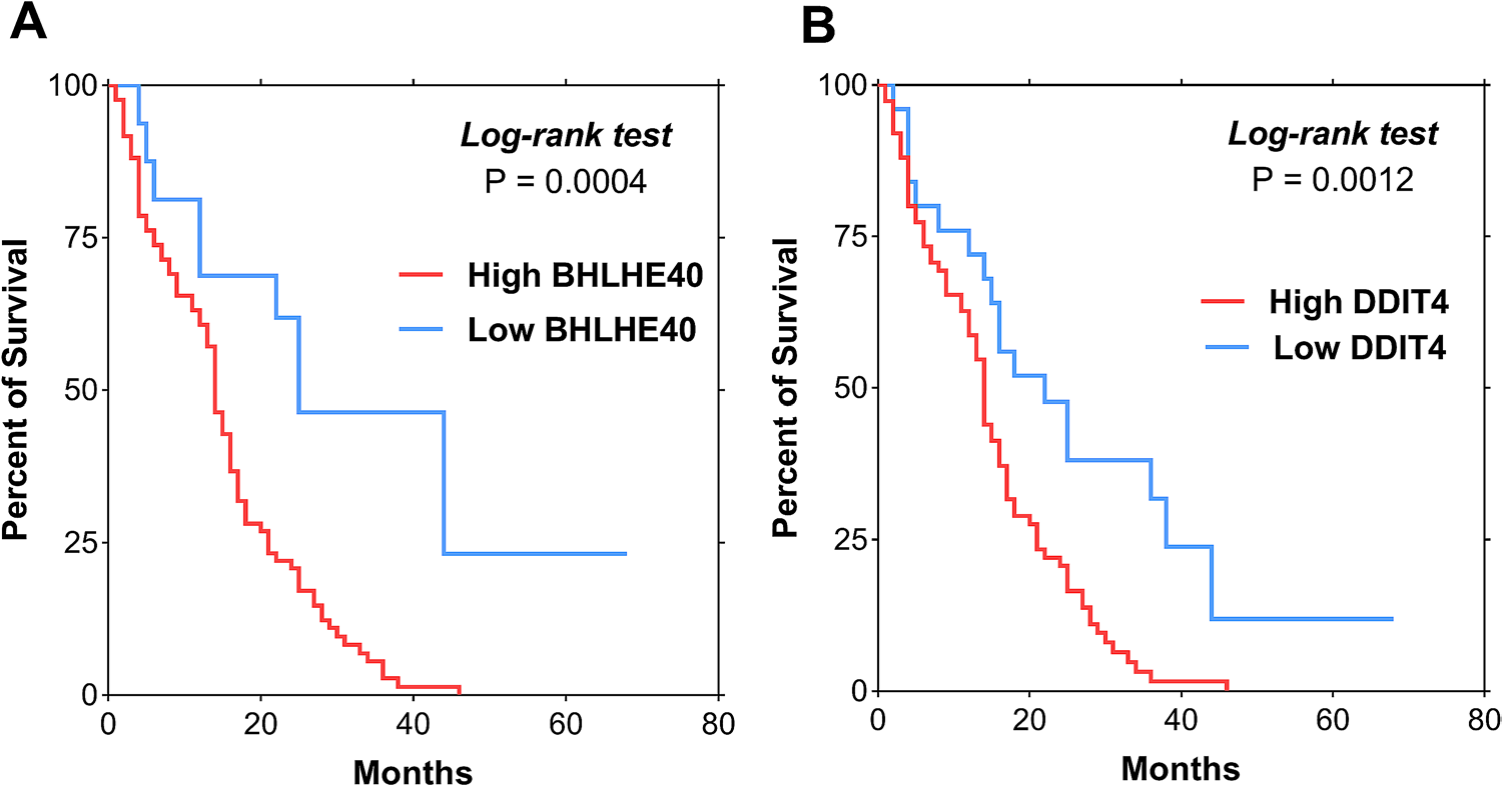
Kaplan–Meier survival curves for overall survival analysis of patients with HBV-HCC (**A**) BHLHE40 (**B**) DDIT4.

The potential prognostic values of BHLHE40 and DDIT4 in terms of overall survival were also analyzed. Based on Kaplan–Meier analysis, the median overall survival of patients with low BHLHE40 levels (< 1.8) was significantly better than that of patients whose levels were ≥ 1.8 (33.8 vs. 15.2 months, *P* < 0.001 by log rank test) (Fig. 6A). Similarly, the median overall survival of patients with low DDIT4 levels (< 2.1) was significantly better than that of patients whose levels were elevated (26.7 vs. 15.0 months, *P* = 0.001) (Fig. 6B).

BHLHE40 and DDIT4 were entered into the multivariate analysis together with other variables that might influence overall survival of the patients. These factors included age, gender, platelet counts, serum TB, AST, ALT, albumin, AFP level, presence of cirrhosis, tumor size and BCLC stage. The multivariate analysis using the Cox proportional hazards model revealed that high BHLHE40 and BCLC stage were independent predictive factors of overall survival. However, DDIT4 was not selected as an independent factor associated with overall survival (Table 2).

**Table 2:** Variables associated with overall survival in patients with HCC.

Variables	Category	Overall survival			
		Univariate analysis		Multivariate analysis	
		OR (95%CI)	*P*	OR (95%CI)	*P*
Age (years)	< 60 vs. ≥ 60	1.02 (0.67–1.55)	0.924		
Gender	Male vs. Female	1.16 (0.66–2.08)	0.608		
Total bilirubin (mg/dL)	< 1.2 vs. ≥ 1.2	0.77 (0.49–1.27)	0.303		
Serum albumin (g/dL)	< 3.5 vs. ≥ 3.5	1.02 (0.63–1.65)	0.946		
Aspartate aminotransferase (IU/L)	< 60 vs. ≥ 60	1.62 (1.06–2.49)	0.027*	1.34 (0.83–2.16)	0.230
Alanine aminotransferase (IU/L)	< 50 vs. ≥ 50	1.63 (1.04–2.53)	0.032*	1.18 (0.73–1.92)	0.493
Platelet count (10^9^/L)	≥ 100 vs. < 100	1.43 (0.85–2.40)	0.178		
Presence of cirrhosis	No vs. Yes	0.97 (0.58–1.64)	0.907		
Alpha fetoprotein (ng/mL)	< 100 vs. ≥ 100	1.93 (1.22–3.05)	0.005*	1.46 (0.88–2.42)	0.146
Tumor size (cm)	< 5.0 vs. ≥ 5.0	1.53 (1.01–2.32)	0.043*	1.09 (0.63–1.90)	0.757
BCLC stage	0-A vs. B vs. C	1.94 (1.33–2.83)	0.001*	2.00 (1.22–3.27)	0.006*
BHLHE40	< 1.8 vs. ≥ 1.8	3.21 (1.59–6.48)	0.001*	3.54 (1.52–8.34)	0.004*
DDIT4	< 2.1 vs. ≥ 2.1	2.29 (1.35–3.91)	0.002*	0.94 (0.48–1.82)	0.850

## Discussion

Recent advances have revealed that genetic and epigenetic alterations accumulated through repeated destruction and regeneration of the hepatocytes are responsible for the development of HCC^14^. In fact, hepatocarcinogenesis is linked to various etiological factors, which in turn result in aberrant activation of different signaling pathways and imbalance between oncogene activation and tumor suppressor gene inactivation. In Thailand, chronic HBV infection is the most important risk factor for HCC development accountable for at least 60% of all cases^15^. Detection of HCC at an early stage is crucial, which allows the possibility of receiving curative treatment and can improve overall survival. Although AFP is currently the most common biomarker for screening HCC in clinical setting, the overall sensitivity and specificity of this tumor marker are approximately 60% and 80%, respectively^16^. Additionally, its sensitivity declines significantly in detecting early HCC because elevated AFP level is typically correlated with large tumor size, poor tumor differentiation and presence of vascular invasion^2^. In our report, for example, approximately 25% of small HCC were AFP-positive (AFP level ≥ 20 ng/mL). Thus, new biomarkers that individually or in complementary with AFP could increase the diagnostic accuracy of early HCC are highly needed.

Given their accessibility, recent data have revealed the alteration of gene expression patterns in PBMCs as a novel source for clinical diagnosis and monitoring in several types of cancers including HCC. In this report, we initially aimed to characterize DEGs of RNA-Seq data derived from PBMCs of patients with HCC compared with healthy controls as a potential diagnostic biomarker of HCC. Additionally, DEGs from co-culture model was generated to mimic human body condition where normal PBMCs were incubated with HCC cell lines. After integrating these results with published microarray database, 24 DEGs with 18 up-regulated and 6 down-regulated genes were identified. Previous data demonstrated that PBMCs from patients with HCC shared distinct and similar features of gene expression profiles with HCC-infiltrating mononuclear inflammatory cells in the liver^9^. Similarly, a recent report also demonstrated a concordance of transcriptomic analysis between PBMCs and tumor tissues in colorectal cancer^17^. In this context, our results indicate transcriptomic changes in PBMCs induced by HCC could represent a readily accessible biomarker for the tumor microenvironment.

Through functional analysis, DEGs identified from our data integration were shown to be associated with various biological processes, including cytokine activity, cell adhesion, inflammatory responses and immune regulation. Additionally, the enriched KEGG pathways of DEGs were mainly involved in TNF signaling, Toll-like receptor signaling, IL-17 signaling, NOD-like signaling pathways and cytokine-cytokine receptor interactions, most of which are closely related to pathogenesis and progression of HCC^14^. For instance, a recent report demonstrated that TNF-α promoted HCC carcinogenesis through the activation and proliferation of hepatic progenitor cells via TNFR2/STAT3 signaling pathway^18^. Regarding the role of IL-17, it was shown that this pro-inflammatory cytokine secreted by Th17 cells could facilitate tumor growth in vitro and in vivo through IL-6/STAT3 pathway in HBV-related HCC^19^. Additionally, previous data suggested that accumulation of intra-tumoral IL-17 accelerated tumor progression through promoting angiogenesis and its detection in cancerous tissues could serve as a potential prognostic marker of HCC^20^.

Among the identified DEGs, 5 candidate cancer-induced genes including BHLHE40, AREG, SOCS1, CCL5 and DDIT4 were selected for external validation on an independent set of PBMC samples of patients with various stages of HCC. The selection of these DEGs was made based on their homogeneous expression patterns in the integrated data set of PBMCs and is known to be involved in pathogenesis of various malignant tumors. According to ROC analysis, the overall results demonstrated that BHLHE40 and DDIT4 expression in PBMCs represented potential diagnostic biomarkers in distinguishing HCC from the non-HCC group. BHLHE and DDIT4 were also found to be specific markers for HCC compared with other cancers such as head and neck cancers and pancreatic cancer.

BHLHE40, also known as DEC1/Stra13/Sharp2, is a stress-responsive transcription factor directly targeted by hypoxia-inducible factor-1α (HIF-1α) in modulating several cell physiological responses^21^. In Addition, BHLHE40 is emerging as a key regulator of immune response during autoimmunity and various inflammatory conditions^22^. Specifically, BHLHE40 has recently identified as a transcriptional regulator of T cell persistence and activity by coordinating metabolic and epigenetic programming, the mechanism of which is critically essential in immunological functions^23^. Current evidence has also indicated that BHLHE40 involve in regulating cell growth, differentiation, proliferation and apoptosis of several cancers^24^. Thus, dysregulation of BHLHE40 could cause alteration of intracellular homeostasis, leading to abnormal cell proliferation, differentiation and subsequent malignant transformation. In breast cancer, it was shown that BHLHE40 constituted an important signaling to promote tumor metastasis by modulating the secretion of epidermal growth factor (EGF), which is known to induce proliferation and invasion of tumor cells under hypoxia^25^. In gastric cancer, BHLHE40 was necessary for anti-apoptotic activity of tumor cells under hypoxic condition by promoting surviving expression^26^. Moreover, BHLHE40 was upregulated in cancerous tissue compared with normal gastric specimens and its expression enhanced during disease progression from well to poorly differentiated, indicating its associated with tumor differentiation status^27^.

With regard to HCC, BHLHE40 was shown to be activated by HIF-1α, suggesting its role in adaptation to a hypoxic microenvironment associated with tumor progression^28^. Indeed, adaptation to hypoxia represents a crucial step in the expansion and transformation of rapidly proliferative cancerous cells, including HCC. A systematic review with meta-analysis has revealed that HIF-1α overexpression is correlated with poor prognosis and aggressive clinicopathological features of HCC^29^. Moreover, it was previously showed in experimental models that overexpression of BHLHE40 promoted epithelial-mesenchymal transition (EMT) process that enhanced metastatic capability of the cancer^30,31^A previous study also reported that higher BHLHE40 expression was detected in cancerous tissue compared with adjacent normal tissues and might be associated with histological differentiation of HCC^32^. Although these available data suggest that BHLHE40 might be participating in HCC development and progression, they were mostly conducted in cell line and tissue-based experiments. However, the role of circulating BHLHE40 expression as a diagnostic and prognostic biomarker in patients with HCC remains to be explored.

In this study, we demonstrate for the first time that BHLHE40 expression in PBMCs could be used as a promising biomarker for HCC. In particular, BHLHE40 was accurately discriminative of AFP-negative and early HCC and its diagnostic performance was more superior than AFP. These findings indicate the potential use of BHLHE40 as a sensitive biomarker for early HCC, as well as a complementary biomarker with AFP-negative HCC in patients with chronic HBV infection. Regarding its predictive role, Kaplan–Meier analysis showed that BHLHE40 overexpression was positively correlated with poor overall survival in patients with HCC. Additionally, multivariate analysis confirmed that an increased BHLHE40 levels in PBMCs was an independently unfavorable predictor of overall survival. Together, our data provide evidence supporting a novel role of circulating BHLHE40 expression in early detection and prognostic indicator of HCC. Given its strong link to adverse clinical outcome, our results might also suggest that targeting BHLHE40 and its related signaling pathways could be a potential therapeutic approach for HBV-related HCC. The DNA damage inducible transcript 4 (DDIT4, also known as REDD1 or RTP801), ubiquitously expressed at low levels in most human tissues, is induced by several transcription factors in response to various stress stimuli such as hypoxic conditions, metabolic alteration and chronic inflammation^33,34^. Dysfunction of DDIT4 has been shown to be associated with multiple disorders including various types of cancers^35^. It was shown that DDIT4 over-expression provided an advantage on cancer cell survival and metastasis in hypoxic conditions through decreased energy consumption, leading to cancer progression, angiogenesis and resistance to chemotherapy or radiotherapy^36^. A recent in silico analysis demonstrated that high levels of DDIT4 were significantly associated with poor prognosis of hematologic malignancies and several solid tumors, such as breast, colon and lung cancers^37^. On the contrary, increased DDIT4 expression was associated with an improved prognosis in gastric cancer but was not related to clinical outcome of ovarian cancers^37^. These data apparently indicate that the role of DDIT4 might not be similar among different cancer types and their aggressiveness^35,36^.

Regarding HCC, the potential role of DDIT4 in this type of cancer, especially HBV-related HCC, remains to be determined. Previous data suggested that DDIT4 might be involved in the pathogenesis of HCC, notably through the expression of miR-802 and programmed cell death protein 1 (PD-1)^38^. In our report, we firstly demonstrated that DDIT4 expression was significantly increased in PBMCs of patients with HCC in comparison with the non-HCC group and healthy individuals, suggesting its potential role in HCC carcinogenesis. Of note, DDIT4 was superior to AFP in differentiating early HCC from the non-HCC group, which had a similar trend as observed in BHLHE40. Additionally, high DDIT4 expression was positively correlated with poor survival by univariate analysis. Although its significance was not reached in multivariate analysis. This result might reflect a strong relationship of circulating levels of BHLHE40 and DDIT4 expression demonstrated in our study.

This report had some limitations as being a retrospective study that enrolled relatively small number of patients with HCC. Moreover, this study focused on patients with chronic HBV infection, which might not be relevant to other chronic liver disease, such as chronic HCV infection and fatty liver disease. Additionally, the cut-off values for relative mRNA expressions identified based on the ROC curves could be variable from study to study depending on patient cohorts and laboratory techniques. Finally, the selection of candidate genes for validation using qRT-PCR was based entirely on their relevance to the pathobiology of various cancers, as well as their consistently up-regulated expression in our study. As a result, down-regulated genes identified from RNA-seq analysis, including CCR2 and ANKRD50, were not chosen for further investigation. Despite such limitations, our data demonstrated that circulating BHLHE40 and DDIT4 were differentially expressed in patients with HCC compared to individuals without cancer. Apart from its diagnostic role, circulating BHLHE40 also emerged as an independent prognosis factor of patients with HBV-related HCC. As current knowledge on the role of BHLHE40 and DDIT4 in HCC remains infancy, further studies are needed to confirm our observations and to elucidate the mechanisms by which these genes play important roles in the pathogenesis and aggressiveness of HCC.

## Materials and methods

### Sample collection

Blood samples were obtained in EDTA tube before treatment procedure from patients with HBV-related HCC, who were diagnosed and treated at King Chulalongkorn Memorial Hospital, Bangkok, Thailand between 2018 and 2020. The diagnostic of HCC was based on the imaging studies results of dynamic computed tomography (CT) or magnetic resonance imaging (MRI) in concordance with the American Association for the Study of Liver Diseases (AASLD) guideline^39^. The demographic and clinical characteristics of patients were collected, which included sex, age, liver function tests, serum AFP level and HCC staging classified by the Barcelona Clinic Liver Cancer (BCLC) system^40^. The blood samples were also obtained from healthy controls and chronic HBV-infected patients without evidence of HCC as control groups.

This study was performed in concordance with the Declaration of Helsinki for the participation of human individuals. The written inform consents were obtained from all patients and all health controls involved in the study. The protocols in this study has been approved by the Institute Ethics Committee of Faculty of Medicine, Chulalongkorn University (IRB No. 313/62).

### PBMCs isolation

PBMCs were isolated from fresh EDTA blood tube by Ficoll-Hypaque density gradient centrifugation using Percoll PLUS density gradient media (GE Healthcare) at 1500 rpm for 30 min at room temperature and were then washed 2 times with PBS. PBMCs were resuspended in 10% DMSO in fetal bovine serum and stored at − 80 °C.

### Cell lines and Co-culture

Liver cancer cells (Huh7, JCRB0403) were obtained from Nation Institutes of Biomedical Innovation, Health and Nutrition JCRB Cell Bank (Osaka, Japan). Cells were grown with DMEM medium (Gibco) supplemented with 10% FBS at 37 °C 5% CO_2_ in culture flask. The cells were harvested at 80% confluence by using 0.05% Trypsin with 0.5 mM EDTA and were then washed by using phosphate buffer saline (PBS).

Co-culture liver cancer cells with PBMCs from healthy individuals were performed in Transwell culture six well plates (Costar). The liver cancer cells (10^6^ cells) were seeded into lower with DMEM and were then incubated overnight at 37 °C and 5% CO_2_. PBMCs from 3 healthy individuals (2 × 10^6^ cells) were individually plated on transwell membrane and incubated for 4 h at 37 °C and 5% CO2. Finally, PBMCs were collected for RNA extractions.

### RNA preparation and sequencing

A total RNA was extracted from PBMCs using TRIzol reagent (Gibco) according to manufacturer’s instruction. For RNA-sequencing, concentration of total RNA samples was quantified using Qubit RNA assay kit (Invitrogen) and RNA integrity was accessed by RNA Electrophoresis with the 2100 Bioanalyzer System (Agilent). After that, RNA samples were then performed library preparation and sequencing by Vishuo Biomedical (Vishuo Biomedical, Singapore). For briefly, library preparation was performed by NEBNext Ultra RNA Library Prep Kit (NEB) according to for Illumina RNA library preparation protocol. mRNA was captured and isolated by NEBNext Poly(A) mRNA Magnetic Isolation Module (NEB). Double stand cDNA was synthesized using random primer, ProtoScript II Reverse Transcriptase and Second stand synthesis Enzyme mix. Then, double stand cDNA was repaired and added a A-tail at both ends of cDNA and was followed by a T-A ligation to add adaptors at both ends of cDNA. Each sample was amplified by PCR for 11 cycles using P5 and P7 primers. The PCR products were validated by 2100 Bioanalyzer and quantified by Qubit 2.0 Fluorometer. After that, libraries with different indices were multiplexed and loaded on an Illumina HiSeq sequencer (Illumina). Sequencing data was obtained as 2 × 150 bp paired end. Ultimately, we obtained an average of 25 million read pairs per samples, which ranged from 20 to 37 million reads. The percentage of data above Q30 and mapping with reference genes were more than 90% of reads (Supplementary Table S5).

### Data processing

The paired-end raw reads were performed by FasTQC version 0.11.2^41^ to check overall sequencing quality and were then trimmed by Trimmomatic version 0.32^42^ to remove the sequencing adaptor and low quality of sequences (lower than Q30). The trimmed reads were aligned to the Homosapiens reference genome (GRCh38) with HISAT2 version 2.1.0^43^ software. Aligned reads were assembled into transcripts by StringTie version 1.3.3b^44^. Then DESeq2 version 1.16.1^45^ was performed to identify DEGs between PBMCs from patients with HCC and healthy controls, as well as PBMCs from co-culture with HCC and without HCC. The up-regulated and down-regulated genes of DEGs were filtered by setting cut-off at 1.5-foldchange and *P*-value ≤ 0.05. The function of DEGs was classified in terms of Gene Ontology (GO) by gProfiler with default settings^46^. The DEGs were then used for cross comparing with microarray data by CU-DREAMX.

### Retrieving data from GenBank and CU-DREAMx analysis

Gene expression profiles of PBMCs from patients with HCC (GSE 49515 and 58208)^12^ were used to compare with RNA-seq data of our study by CU-DREAMX. Briefly, DEGs in PBMCs of these datasets derived from 38 patients with HCC cases and 14 healthy controls were identified (*P* < 0.05) and then compared with DEGs in PBMCs from patients with HCC and co-culture model to identify the intersection of DEGs among three datasets. Additionally, the intersect DEGs in PBMCs of HCC from CU-DREAMX were compared with DEGs in PBMCs from 27 patients with head and neck cancers (GSE39400) and 3 patients with pancreatic cancer (GSE49515).

### Quantitative RT-PCR analysis

The performance of identified DEGs was further validated in PBMCs of patients with HBV-related HCC and non-HCC individuals by qRT-PCR. The criteria for selecting candidate genes based on the consistency of up-regulated expression in PBMCs of patients with HCC from RNA-seq and known to be involved in pathogenesis of various malignant cancers. In this regard, BHLHE40, AREG, SOCS1, CCL5 and DDIT4 genes were selected. The total RNA was extracted from PBMCs of 100 patients with HBV-related HCC, 100 patients with chronic hepatitis B and 100 healthy controls using TRIzol reagent (Thermo Scientific). Then, cDNA was synthesized using RevertAid First Strand cDNA Synthesis (Thermo Scientific). The qRT-PCR reaction contained 6.25 µL of QPCR Green Master Mix HRox 2 × (Biotechrabbit), 0.25 μL of primers and 1 μL of cDNA and nuclease-free water in a total volume of 12.5 μL. The reactions carried out on a QuantStudio 5 Real-Time PCR System (Applied Biosystems). Primer sequences and thermal cycle condition are presented in Supplementary Table S6. All reactions were performed in duplicate. Positive controls for each target genes and negative controls were included to ensure correct interpretation. The expression of target genes was normalized by β-globin endogenous reference gene. The data are shown in log_2_fold change format.

### Statistical analysis

Statistical analysis was performed using the Statistical Package for the Social Sciences (SPSS) version 23 (https://www.ibm.com/analytics/spss-statistics-software) and GraphPad prism version 8 for Window (https://www.graphpad.com/scientific-software/prism). Comparisons between groups were analyzed by Chi’s square or Fisher’s exact test for categorical variables and by Student’s t-test or one-way ANOVA for quantitative variables. Spearman's rank test was used for correlations between parameters. Kaplan–Meier analysis and log-rank test were used for survival analysis. The Cox regression analysis was conducted to identify independent factors associated with overall survival of patients with HCC. *P-*value < 0.05 was considered statistically significant. Adjusted *P*-value for the multiple hypothesis testing was not performed in this study due to small number of samples. However, we further validate the results with highly sensitive method using qPCR in the independent cohort.

## Supplementary Information

Supplementary Figure S1.

Supplementary Figure S2.

Supplementary Figure S3.

Supplementary Figure S4.

Supplementary Figure S5.

Supplementary Tables.

Supplementary Legends.
