# Supplementary figures and images for: Identification of BHLHE40 expression in peripheral blood mononuclear cells as a novel biomarker for diagnosis and prognosis of hepatocellular carcinoma
Source: Sci Rep. 2021 May 27;11:11201. doi: 10.1038/s41598-021-90515-w (PMC8159962; doi:10.1038/s41598-021-90515-w)

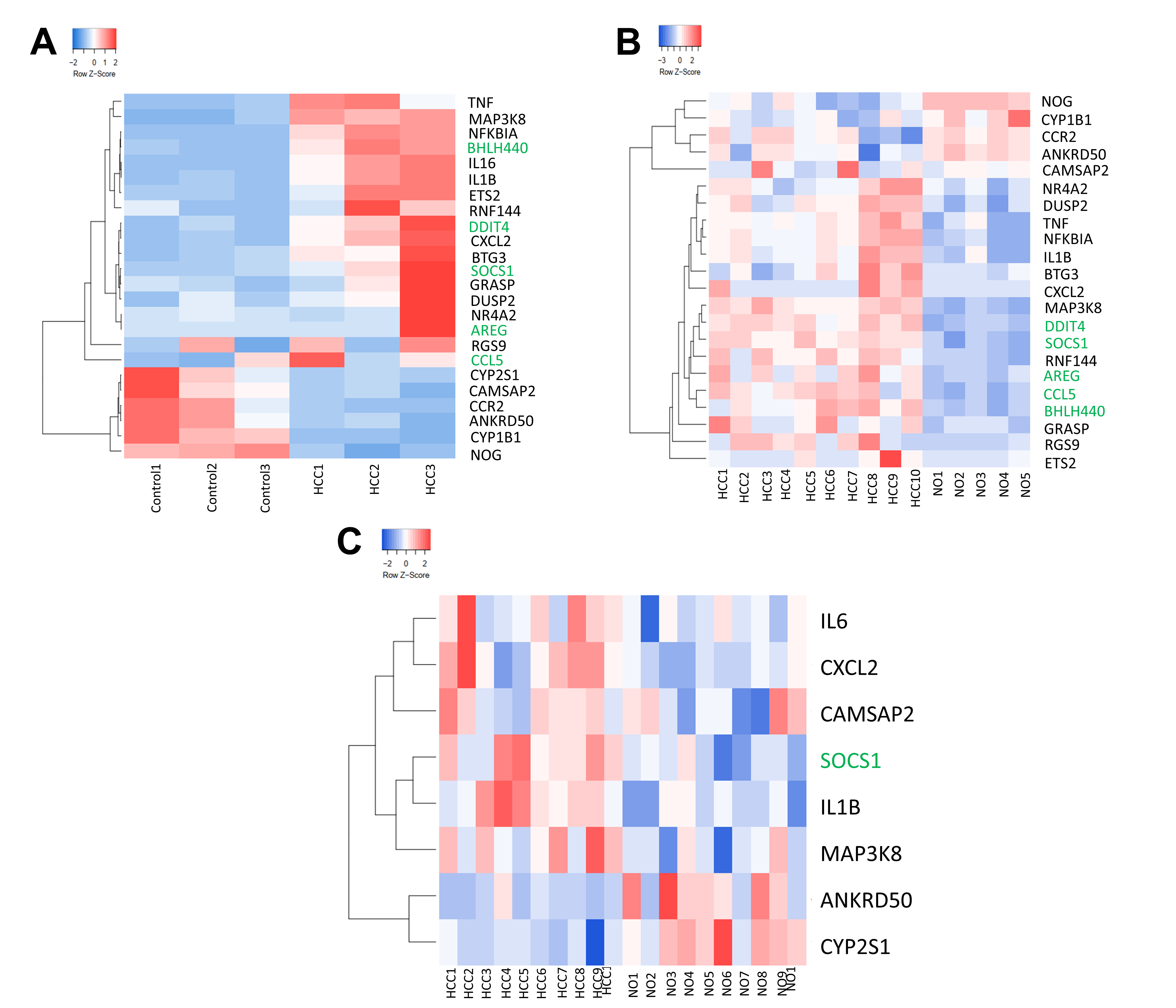

Supplement: Supplementary file 1 — Supplementary Figure S1. [file 41598_2021_90515_MOESM1_ESM.tif]

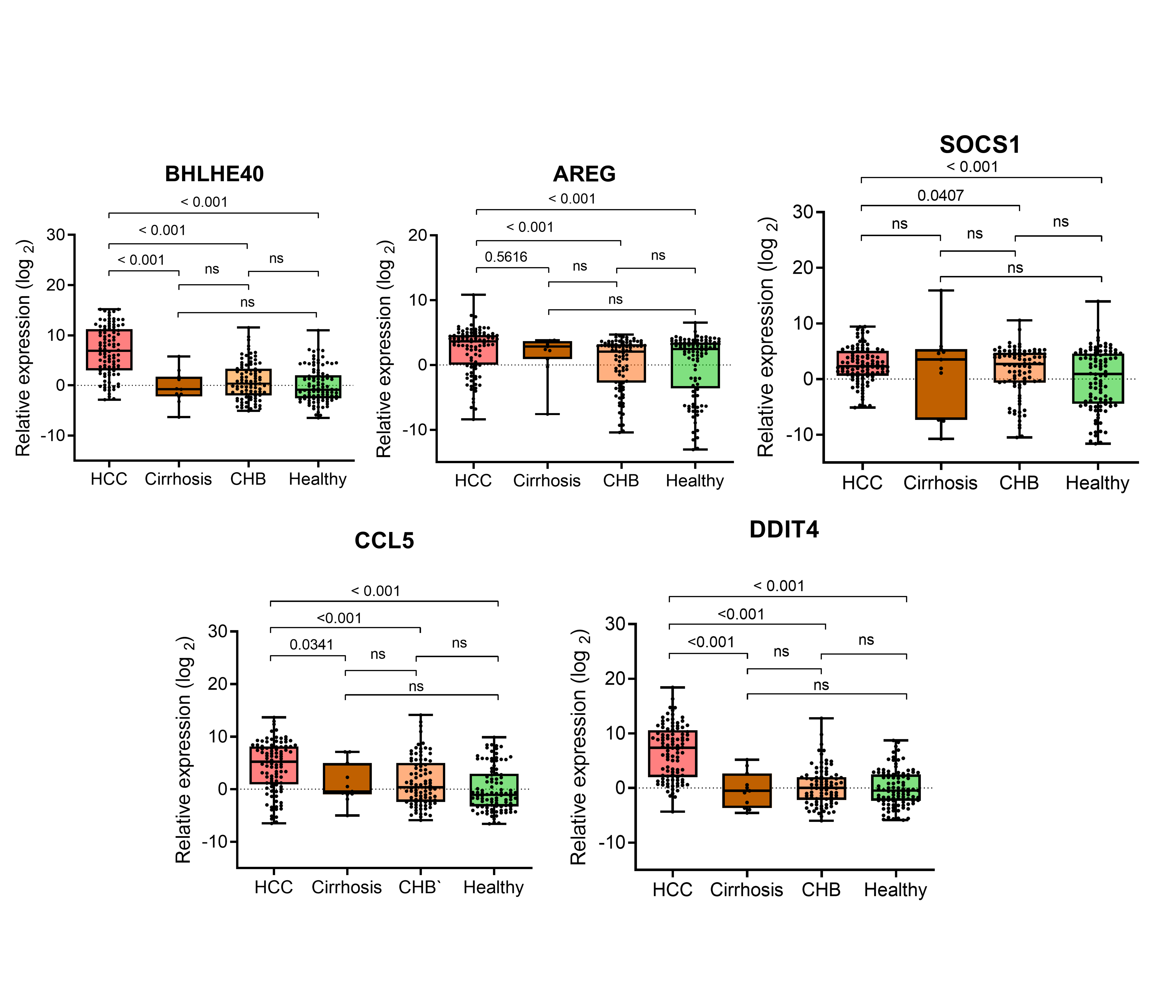

Supplement: Supplementary file 2 — Supplementary Figure S2. [file 41598_2021_90515_MOESM2_ESM.tif]

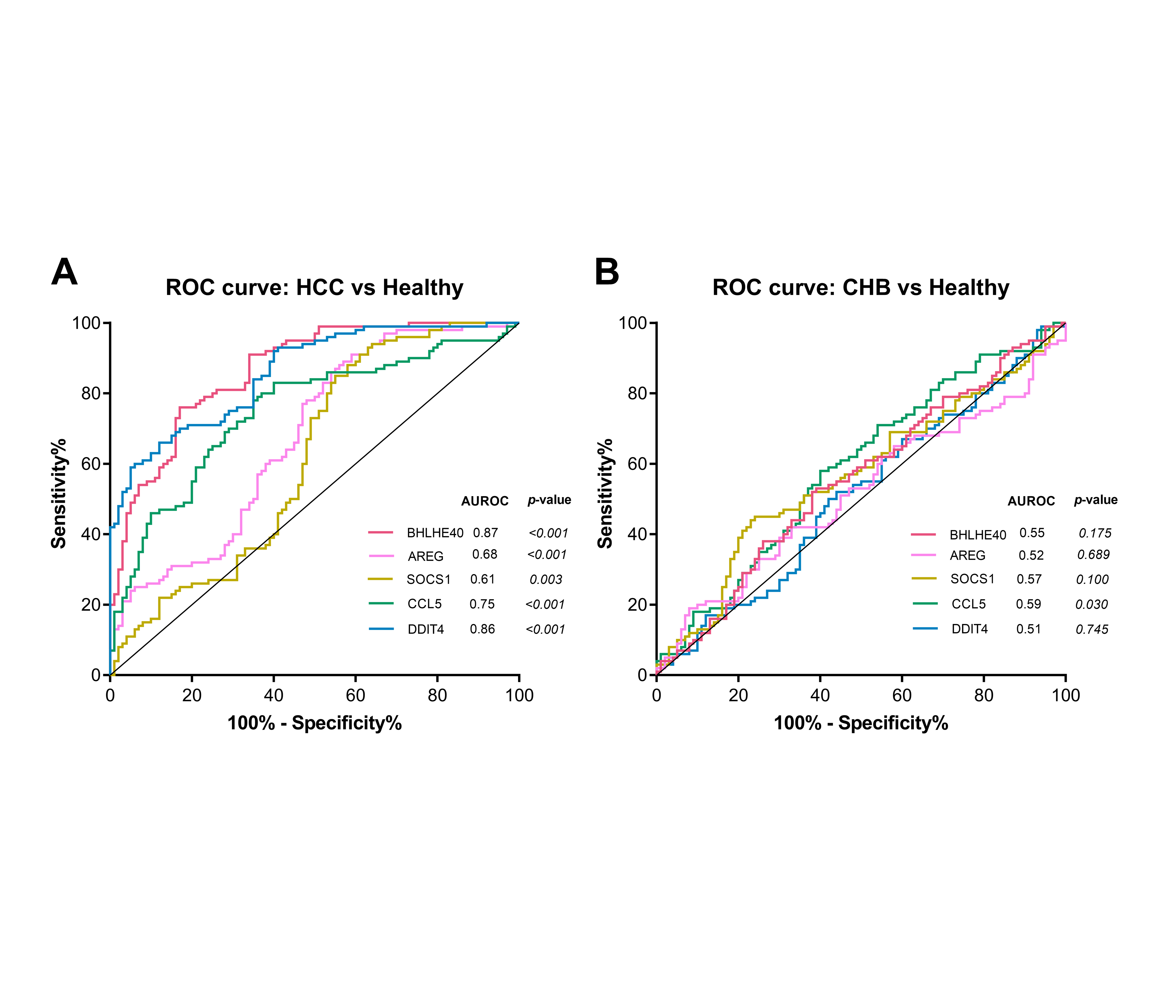

Supplement: Supplementary file 3 — Supplementary Figure S3. [file 41598_2021_90515_MOESM3_ESM.tif]

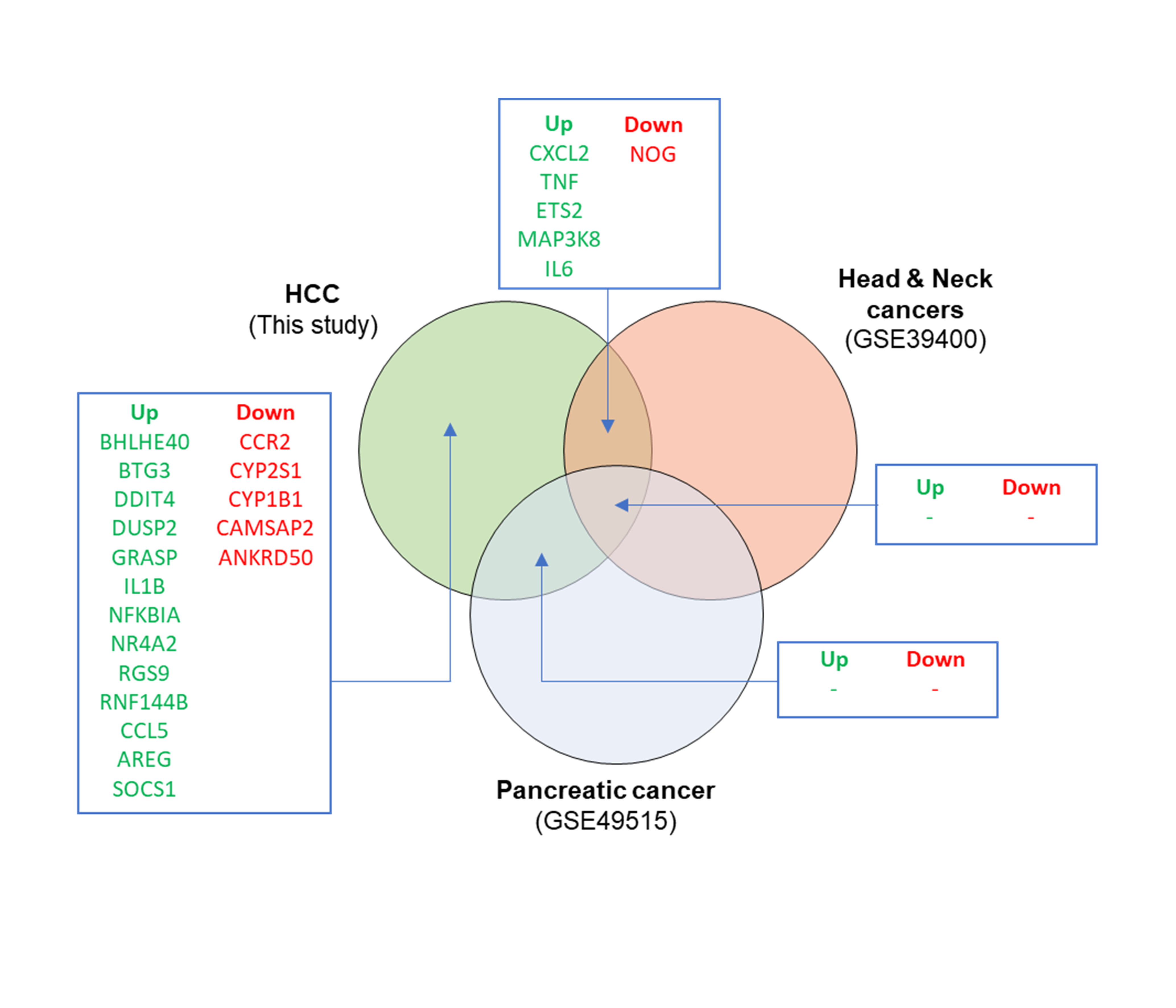

Supplement: Supplementary file 4 — Supplementary Figure S4. [file 41598_2021_90515_MOESM4_ESM.tif]

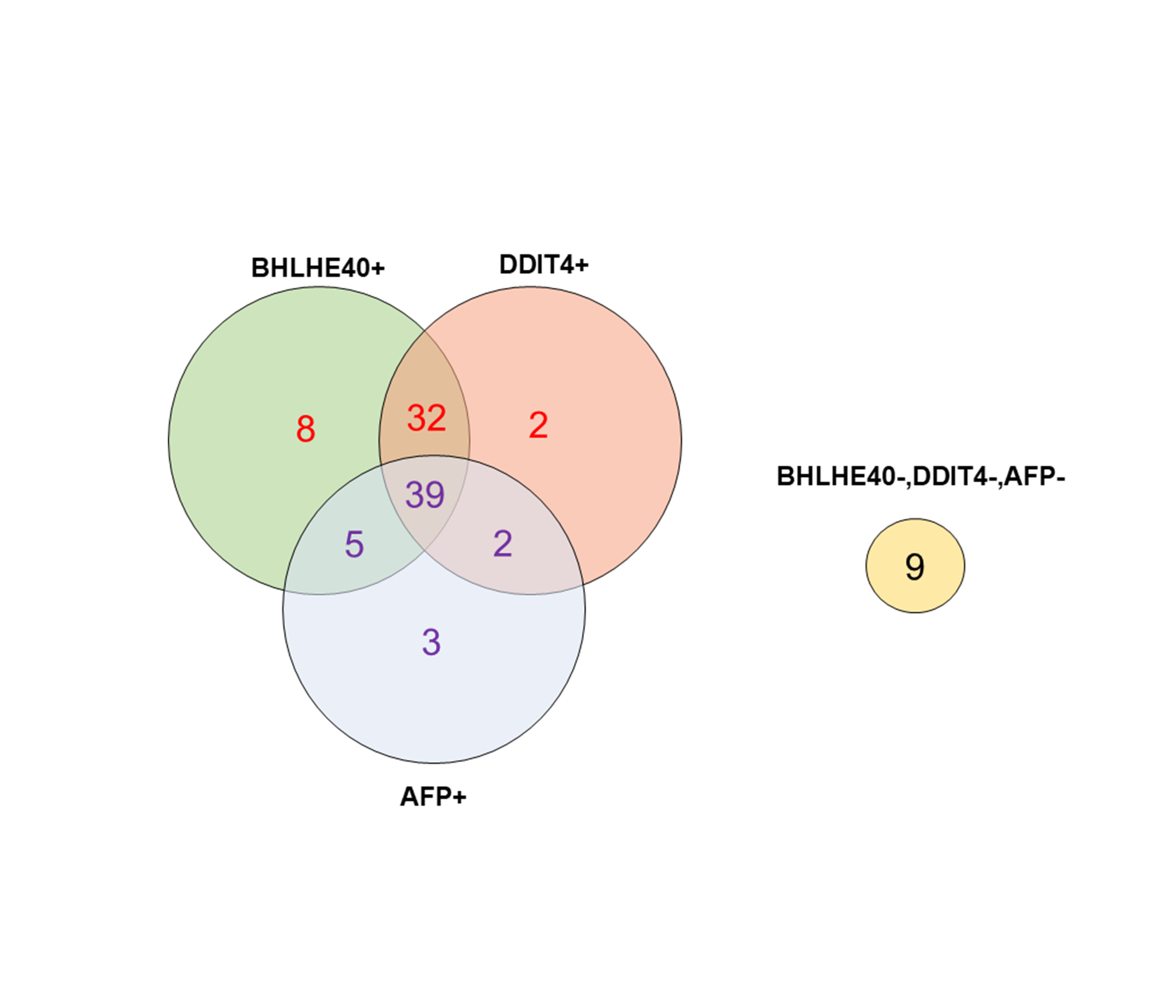

Supplement: Supplementary file 5 — Supplementary Figure S5. [file 41598_2021_90515_MOESM5_ESM.tif]
