# Supplementary material for: Identification of BHLHE40 expression in peripheral blood mononuclear cells as a novel biomarker for diagnosis and prognosis of hepatocellular carcinoma
Source: Sci Rep. 2021 May 27;11:11201. doi: 10.1038/s41598-021-90515-w (PMC8159962; doi:10.1038/s41598-021-90515-w)
Supplement: Supplementary file 7 — Supplementary Legends. [file 41598_2021_90515_MOESM7_ESM.docx]

**Supplementary information**

**Figure S1.** Heatmap of cancer-induced genes expression. (**A**) Co-culture model, (**B**) GSE58208 and (**C**) GSE49515. Candidate of cancer-induced genes for validation are labels in green.

**Figure S2.** Relative expression of candidate cancer-induced genes in PBMCs of patients with HCC, cirrhotic-CHB, non-cirrhotic CHB and healthy controls. (A) BHLHE40, (B) AREG, (C) SOCS1, (D) CCL5 and (E) DDIT4. Relative expression of genes represents as log_2_ on y–axis.

**Figure S3**. Receiver operating characteristic (ROC) curves of the cancer-induced genes of PBMCs in differentiating (A) patients with HCC and healthy controls, (B) patients with CHB and healthy controls

**Figure S4.** Venn diagram of intersect genes between candidate genes in this study and other cancers from GSE49515 and GSE39400.

**Figure S5.** Venn diagram of positive/negative biomarkers in 100 HCC patients. Patients with positive of BHLHE40 and/or DDIT4 but not AFP positive are highlighted in red. Patients with positive of AFP are highlighted in purple.

**Table S1** Baseline characteristics of patients with HCC and healthy controls for RNA-sequencing

**Table S2** Summary of intersect genes.

**Table S3** Summary of diagnostic performance of cancer-induced genes

**Table S4** Correlation of gene expression and clinical parameters

**Table S5** Summary of reads of RNA sequencing.

**Table S6** Summary of primer sequences and thermal cycle conditions
